# Advances in the assessment of minimal residual disease in mantle cell lymphoma

**DOI:** 10.1186/s13045-020-00961-8

**Published:** 2020-09-24

**Authors:** Dayoung Jung, Preetesh Jain, Yixin Yao, Michael Wang

**Affiliations:** 1 Department of Lymphoma/Myeloma, The University of Texas MD Anderson Cancer Center, 1515 Holcombe Blvd, Houston, TX 77030 USA; 2 Department of Hemapathology, The University of Texas MD Anderson Cancer Center, 1515 Holcombe Blvd, Houston, TX 77030 USA

**Keywords:** Minimal residual disease, Mantle cell lymphoma, ctDNA, Liquid biopsy, Next-generation sequencing

## Abstract

The clinical impact of minimal residual disease detection at early time points or during follow-ups has been shown to accurately predict relapses among patients with lymphomas, mainly in follicular and diffuse large B cell lymphoma. The field of minimal residual disease testing in mantle cell lymphoma is still evolving but has great impact in determining the prognosis. Flow cytometry and polymerase chain reaction-based testing are most commonly used methods in practice; however, these methods are not sensitive enough to detect the dynamic changes that underline lymphoma progression. Newer methods using next-generation sequencing, such as ClonoSeq, are being incorporated in clinical trials. Other techniques under evolution include CAPP-seq and anchored multiplex polymerase chain reaction-based methods. This review article aims to provide a comprehensive update on the status of minimal residual disease detection and its prognostic effect in mantle cell patients. The role of circulating tumor DNA-based minimal residual disease detection in lymphomas is also discussed.

## Background

**Fig. 1:**
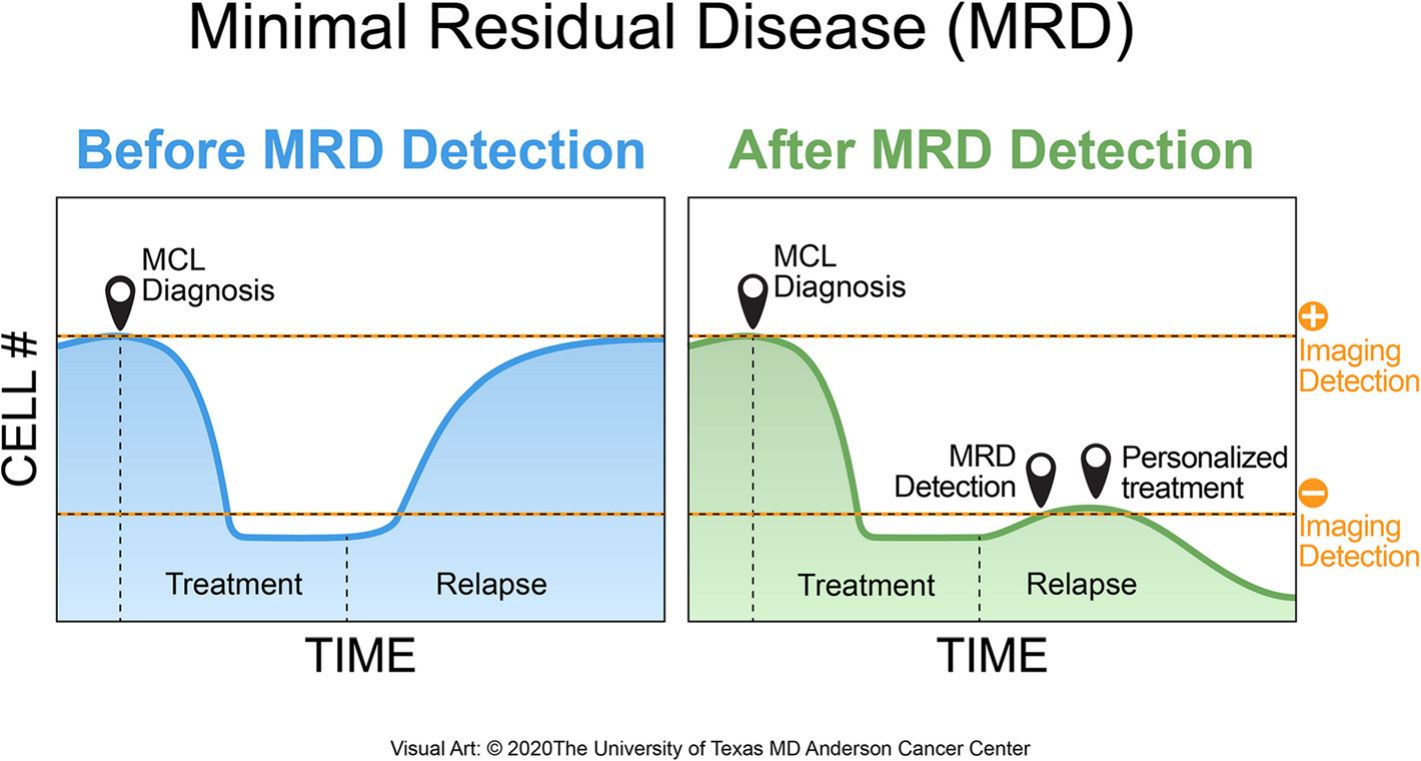
General overview of minimal residual disease detection. The figure shows two scenarios emphasizing the importance of MRD detection after initial treatment of mantle cell lymphoma. When MRD detection is not performed, there is no indication of how effective the treatment was on the tumor, and relapse may eventually occur (left). If MRD diagnosis confirms a positive result, the patient can be prescribed to a more personalized treatment to prevent any future relapses (right)

Minimum residual disease (MRD) in lymphoma refers to the presence of a minimal burden of clonal lymphoma cells without apparent signs or symptoms of the disease [1]. Residual cancer can persist in either the bloodstream as circulating tumor cells or can be tracked in tissue sites as circulating tumor DNA, making MRD detection possible via peripheral blood (PB) or bone marrow (BM) samples [2, 3]. These persistent cancer cells can predict relapse or recurrence of tumors in almost all cancer types, making MRD status a good prognostic marker (Fig. 1). MRD detection methods have been widely studied in patients diagnosed with chronic lymphocytic leukemia (CLL) and now in large B cell lymphomas. These studies identified MRD to be a strong independent prognostic marker predicting disease recurrence [1, 4–6]. Thus, it is important for these methods to be highly sensitive to detect lowest levels of clonal cells and provide clinicians with a valuable prognostic method, possibly aiding in better clinical management. As such, the detection of residual disease has proven beneficial for patient management. MRD detection is especially important in non-Hodgkin’s lymphomas (NHLs) as a prognostic factor, including diffuse large B cell lymphoma (DLBCL), follicular lymphoma (FL), and mantle cell lymphoma (MCL) [7]. Although MCL only represents about 6% of all NHL cases, it is one of the most aggressive subtypes with a median overall survival being around 8 years [8]. MCL’s genetic hallmark is the chromosomal translocation t(11;14)q(13;32), which ultimately leads to an overexpression of cyclin D1, and is considered heterogeneous in both biological and clinical aspects [9]. While new treatment strategies have shown promising results, resistance after ibrutinib is being noted, making it even more difficult for patients to attain a durable complete response [8]. Unlike other lymphoma subtypes, MCL significantly invades both PB and BM in over 90% of cases, making it more advantageous to detect MRD [10].

Positron emission tomography and computed tomography imaging are commonly utilized to assess disease burden among cancer patients. However, these imaging methods come with many limitations as they are not able to capture dynamic processes that are inherent in the progression of NHL subtypes, such as clonal evolution and tumor response kinetics [1, 11, 12]. They are not able to detect any residual disease either, which have shown to be predictive of future relapse. Because imaging methods cannot detect MRD, techniques such as flow cytometry (FC)- and polymerase chain reaction (PCR)-based detection platforms have become the main types for MRD diagnostics. The MRD concept has been developing for the past 20 years in the context of MCL, initially based on evidence from PCR-based methods [13]. However, these methods also have major drawbacks as they cannot accurately capture the tumor progression. Thus, alternative and more sensitive methods have emerged, which can be characterized into a single category: next-generation sequencing (NGS). Although NGS methods have been on the rise in recent years, real-time quantitative PCR (qPCR) is still considered to be the gold standard as the clinical utility of NGS methods still needs prospective evaluation [14]. Because of this, qPCR has been compared with alternative methods in many studies [1, 15, 16]. Since NGS methods could have implications to change existing practices in detecting MRD in MCL, it is crucial to acknowledge the different methods, their strengths and limitations, and most importantly, their applications in the clinical setting (Table 1). This assessment aims to provide a comprehensive and comparative review of currently available MRD detection methods and to shed light on recent improvements, challenges, and applications for determining relapses in patients with MCL.

**Table 1:** Overview of the available MRD detection methods in MCL

Feature	FC	PCR		NGS	
Name	*MFC*	*qPCR*	*ddPCR*	*IgNGS*	*mutNGS*
Target	Immunophenotype	IgH rearrangement, *t(11;14), SOX11, CCND1*	Somatic mutations	IgH rearrangement	Somatic mutations
Detection limit	4-color: 10^-3^ 8-color: 10^-4^, 10^-5^	10^-5^	10^-5^	10^-6^	10^-6^
Turnaround time	3–4 h	2 weeks	Less than a week	1 week	1–2 weeks
Strengths	-Rapid quantification -Availability	-Well validated with low calibration failure -Robust and accurate -High sensitivity -High reproducibility -Regular quality control rounds	-Fast -Theoretical potential for increased sensitivity for low-level clones -Absolute quantification method	-High sensitivity -Can detect MRD not identified by multi-parameter FC or qPCR -Independent of patient-specific primers -Availability -Fast due to universal reagents	-Applicable to all lymphoma subtypes -Liquid biopsy -Broad genomic information -Can track clonal evolution and resistance
Limitations	-Comparatively not sensitive -Not standardized -Requires presence of circulating tumor cells -Requires expertise in analysis	-Time consuming -Primer design necessary -Non universal reagents -Assessment limited to few genetic lesions -Cannot track clonal evolution -Labor intensive for tumors without canonical translocations -Not an absolute quantification tool	-Limited capability for multiplexing -Tracking of clonal evolution limited by throughput -Requires specialized technology not widely accessible	-ctDNA detection cannot identify the site or extent of disease relapse -Limited genomic information as it only allows Ig sequencing -Only tracks Ig-based clonal evolution -Primary sample required -Complex bioinformatics evaluation -Requires tumor tissue for clonotype determination	-Limitation in sensitivity due to breadth of genes and sequencing depth -Limited detection at low allele frequencies

## Current status of MRD detection in mantle cell lymphoma

### Commonly used MRD markers in MCL

The two commonly targeted MRD markers in MCL are the immunoglobulin heavy chain (IgH) rearrangements and the Bcl1-IgH rearrangement derived from t(11;14) (q13;q32). The IgH gene is located at 11q32 on chromosome 14. Of the 11 IgH genes, nine are functional, corresponding to the nine isotypes of the heavy chain [17]. B cells’ early differentiation takes place in the BM, where the variable (V), diversity (D), and joining (J) gene segments of the IgH undergo rearrangement. The germ-line DNA rearranges to generate a functional repository of cells, capable of producing antibodies that have diverse functions in the human body. While the production of various antibodies via VDJ rearrangement provides essential immune system capacity, it also leaves an opportunity for malignant transformation through the translocation of a proto-oncogene [17]. Therefore, amplifying the genomic tumor DNA from patients using IgH locus-specific primers, followed by IgH rearrangement sequencing, is a very useful tactic to detect specific clones (i.e., clonotypes). Alternatively, the recurrent cytogenetic aberration indicated by the chromosomal translocation t(11;14) can also serve as a valuable MRD marker. Here, the IgH locus at 14q32.3 is involved and the Bcl1 region from 11q13 is translocated into the IgH locus. This translocation also involves VDJ recombination, resulting in junctional regions that are unique. Another approach to detect MRD involves identifying the landscape of somatic mutations in the tumor cells. Since tumor cells divide rapidly, they form numerous clones harboring the same mutations. Therefore, with a priori knowledge of somatic mutations in the tumor, one can develop a library, amplify the mutations using gene-specific primers among patients’ specimens, and subsequently sequence the product to detect MRD levels [1].

### FC-based MRD methods

Flow cytometry uses fluorescent-labeled antibodies to identify different cell populations according to size (light scatter) and immunophenotype (fluorescence emission), enabling identification of both circulating and BM tumor cells [1]. Although multi-parameter flow cytometry (MFC) has been tested in acute lymphocytic leukemia for levels above 0.01% and has shown MRD to be an independent prognostic factor in CLL, the same does not always apply to MCL [10, 18]. When comparing different MRD detection methods in MCL, the MFC method has a detection limit of only 8 × 10^-4^ within both PB and BM samples [19]. Panels containing a range of antibodies can be utilized in FC methods, with a 4-color panel being the most commonly available but with the lowest sensitivity for MRD quantification [10]. MFC using extra colors have been associated with a more robust sensitivity of at least 0.01% and is clinically relevant and feasible in managing individualized therapy [10]. The 8-color MFC was used alongside a PCR method to assess MRD in a study involving MCL patients treated with ibrutinib and venetoclax. MRD clearance was confirmed in 67% of patients using this method and among patient samples who achieved complete response, 14 out of 15 eligible samples showed a negative status [20] (Table 2). When MFC was created similarly to the standardized CLL protocol to measure MRD using selected surface antigens on MCL cells, those possessing a combination of CD20/23/5/19/200/62L/3/45 was the most favorable in MFC measurement, albeit at a sensitivity of only 2 × 10^-4^. This exemplifies the importance of an appropriate target for MRD [34]. Although FC methods possess many advantages, such as being fast and readily available, a need for improved strategies using novel antibody combinations is still required to achieve a comparable sensitivity to the gold standard [10, 34]. This is especially true for MCL due to its heterogeneity, which requires widespread marker combinations. A major drawback of MFC is the requisite of a cell surface marker pattern specific to MCL at a high-enough level (over 10^-5^ to 10^-4^). Even at the time of diagnosis, the circulating tumor burden is often too low for MRD detection to occur [7].

**Table 2:** Summary of MCL studies using MRD

Author	MRD method/Target	Population	Treatment type	MRD results
Cowan et al. [4]	qPCR and MFC/IgH rearrangement or t(11;14) translocation	Previous ASCT MCL pts in clinical CR	ASCT	MRD positivity is independently associated with poor outcomes after ASCT for MCL patients in CR
Liu et al. [6]	qPCR/IgH rearrangement	Treatment-naïve MCL pts	R-CHOP +ASCT*	MRD negativity: 46% (74%*)
Tam et al. [20]	MFC and ASO-qPCR/IgH rearrangement or t(11;14) translocation	R/R MCL pts	Ibrutinib and venetoclax	MRD negativity: -MFC 67% -ASO-qPCR 38%
Klener et al. [21]	qPCR/IgH rearrangement	Transplant-ineligible MCL pts who received R-CHOP/R-cytarabine	R-CHOP/R-cytarabine + rituximab maintenance	MRD (positive vs negative) after 3 or 6 cycles of induction therapy not correlated with PFS
Hermine et al. [22]	ASO-qPCR/not specified	Treatment-naïve MCL pts	R-CHOP + ASCT* (control) vs R-CHOP/R-DHAP + ASCT* (cytarabine)	MRD negativity: -PB: 47% in control and 79% in cytarabine (68%* vs 85%*) -BM: 26% in control and 61% in cytarbine (59%* vs 68%*)
Kolstad et al. [23]	Nested PCR/IgH rearrangement	Treatment-naïve MCL pts	R-HDS +ASCT*	MRD negativity: 56% (86%*)
Albertsson-Lindblad et al. [24]	Nested PCR/IgH rearrangement	Treatment-naïve MCL pts	Lenalidomide + bendamustine + rituximab	MRD negativity: -After 6 cycles 36% -Therapy completion: 64%
Kolstad et al. [25]	Combined nested and qPCR/IgH rearrangement	ASCT and PCR marker available MCL pts	ASCT Possible rituximab maintenance	58 pts experienced MRD relapse and got rituximab maintenance. This converted pts to MRD-(87%) but many became positive again later (69%)
Starza et al. [26]	Nested and qPCR/IgH rearrangements	From 4 labs of the FIL MRD Network: Both MCL and FL pts	N/A	156/187 samples concordantly classified as MRD positive or negative by both methods; 31 samples were borderline
Szostakowska et al. [27]	qPCR/t(11;14), IgH rearrangement, *SOX11*	MCL pts at diagnosis and treatment	Various	At diagnosis: high *SOX11* had shorter PFS vs low *SOX11* (*p* = 0.04)
Drandi et al. [28]	qPCR, MFC, ddPCR/IgH rearrangement	MCL MRD samples from 4 prospective trials of the European MCL Network	N/A	All 3 methods gave comparable results in MRD samples with 0.01% positivity. ddPCR was preferable to qPCR on BQR samples.
Armand et al. [29]	ClonoSeq/IgH rearrangement	Treatment-naïve MCL pts	Rituximab + bendamustine / Rituximab + cytarabine	MRD negativity: 93%
Ruan et al. [30]	ClonoSeq/IgH rearrangement	Treatment-naïve MCL pts	Lenalidomide + rituximab	MRD negativity: 8 out of 10 subjects
Ladetto et al. [31]	qPCR and NGS/IgH rearrangement	ALL, MCL, and MM pts	N/A	NGS had same level of sensitivity as ASO-qPCR
Smith et al. [32]	ClonoSeq and MFC/IgH rearrangement	Treatment-naïve MCL pts	Bendamustine-rituximab + rituximab ± lenalidomide	MRD negativity: -NGS: 91% PB and 90% BM -MFC:95% PB
Callanan et al. [33]	qPCR/IgH rearrangement	MCL pts < 66 years of age	R-DHAP +ASCT* + rituximab maintenance	MRD negativity: -80% ➔ 95%* PB -66% ➔ 82%* BM

*qPCR* real-time quantitative PCR, *MFC* multiparameter flow cytometry, *IgG* immunoglobulin heavy chain, *ASCT* autologous stem cell transplant, p*ts* patients, *CR* complete response, *R-CHOP* rituximab/cyclophosphamide/doxorubicin hydrochloride/vincristine sulfate/prednisone, *R/R* relapsed/refractory, *PFS* progression-free survival, *PB* peripheral blood, *BM* bone marrow, *R-HDS* rituximab/high-dose sequential chemotherapy, *FL* follicular lymphoma, *ddPCR* digital droplet PCR, *BQR* below the quantitative range samples, *ALL* acute lymphocytic leukemia, *MM* multiple myeloma, *NGS* next-generation sequencing, *R-DHAP* rituximab/dexamethasone/cytarabine/cisplatin

### PCR-based MRD methods

**Fig. 2:**
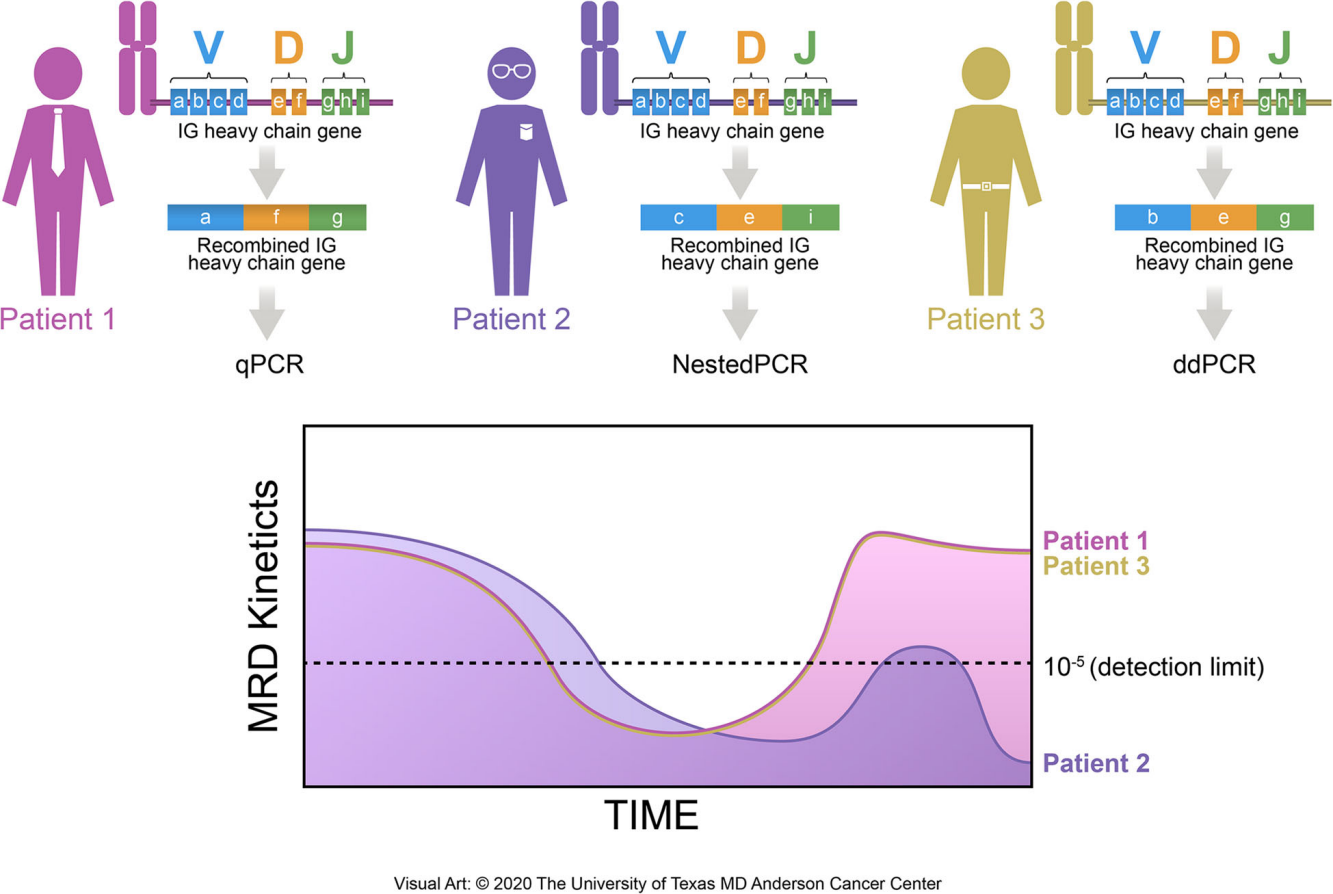
PCR-based MRD detection methods. qPCR is the gold standard method that uses IgH rearrangements as clonal markers. Nested PCR uses double amplification and considered to be more qualitative. ddPCR is an absolute quantification method that overcomes many of qPCR’s limitations. In this hypothetical situation where each MCL patient undergoes MRD assessment via different PCR methods, nested PCR is not able to meet the quantitative standards and thus, is less sensitive compared with the qPCR and ddPCR

By detecting tumor-specific DNA sequences and chromosomal rearrangements, PCR methods can amplify these targets via specific primer designs for a gene of interest. This method works well in different NHLs, as each tumor contains a monoclonal IgH that can serve as the measure of lymphoma cells. There are generally two PCR-based MRD detection techniques: qPCR and digital droplet PCR (ddPCR) (Table 1). As the most well-known and exploited technique in the detection of MRD in MCL, qPCR uses either IgH rearrangements or the Bcl1-IgH rearrangement as its clonal markers and has a high sensitivity and ability to detect 80–95% of MCL (Fig. 2) [1, 19]. In a study that looked at the prognostic impact of MRD following induction immunochemotherapy (rituximab, methotrexate, and augmented CHOP) and before high-dose consolidation among 39 MCL patients, qPCR was successful in detecting both IgH and Bcl-1/JH gene rearrangements. MRD detection predicted disease progression with a hazard ratio of 3.7 (*p* = 0.016), making it an independent predictor for these patients [6]. This prognostic value of MRD status between induction immunochemotherapy and high-dose consolidation has also been seen when evaluating if MRD at this time point could stratify outcomes for patients in complete remission from MCL. Among 75 MCL patients, the MRD-positive status was indeed associated with both shorter overall survival (OS) and progression-free survival (PFS) [4]. The same results were not replicated in a more recent study analyzing the prognostic significance of MRD in elderly MCL patients post induction with alternating R-CHOP (rituximab, cyclophosphamide, doxorubicin hydrochloride, vincristine sulfate, prednisone) and R-cytarabine (3 + 3 cycles), followed by rituximab maintenance [21].

Within qPCR is the allele-specific oligonucleotide quantitative PCR (ASO-qPCR), in which the Ig complementarity-determining region is used to generate the patient-specific primers and probes [19, 35]. By doing so, it reveals the distinctiveness of each patient at a sensitivity of 10^-4^ to 10^-6^ [36]. In the MCL Younger trial, ASO-qPCR detected MRD during the first-year post-autologous stem cell transplant (ASCT) within two cohorts: the control (R-CHOP and ASCT) and the cytarabine (alternating R-CHOP and R-DHAP and ASCT) groups. While MRD assessment was not a primary outcome, the results were strongly prognostic for PFS, independent from the MCL International Prognostic Index (MIPI) score [22]. In an aforementioned cohort of patients treated with ibrutinib and venetoclax, ASO-qPCR was applied to PB samples to target either clonal IgH rearrangement or t(11;14) translocation. The presence of MRD was not a primary outcome in this study either, but it did show that among 24 samples, MRD clearance was confirmed in 38% (9 out of 24) of patients posttreatment. Among those who had complete response, 9 out of 11 samples that were eligible for ASO-qPCR MRD detection had a negative status [20].

Nested PCR is also a commonly used method in various MRD studies, albeit being more qualitative than quantitative. It consists of using 2 primer sets and 2 PCR reaction steps, where the first amplification is non-tumor specific and the second is tumor specific [9]. In the Nordic MCL3 study where ^90^Y-ibritumomab-tiuxetan (Zevalin) was given to patients who did not achieve CR after BEAM/C, and before transplant, MRD clearance was assessed via nested PCR. Fifty-six percent of patients achieved MRD negativity before transplant, and the number increased to 86% post-transplant, indicating that high-dose consolidation can contribute to a long-term survival in MCL [23]. When lenalidomide was added to rituximab maintenance in the Nordic MCL4 trial, more than 50% of both BM and PB samples were MRD positive using the standard nested PCR [24]. In the combined Nordic MCL2 and 3 study, the nested PCR method was combined with qPCR to detect Bcl-1/IgH and IgH rearrangements in MCL to guide preemptive treatment with rituximab. This treatment seems to have changed MRD status from positive to negative and delayed clinical relapse [25]. Finally, in an effort to standardize MRD methodologies at the national level, the Fondazione Italiana Linfomi MRD Network collaborated since 2009 and were able to analyze MRD in both FL and MCL patients. When applying both qPCR and nested PCR to assess MRD, there was an 83% concordance between the 2 methods, while the rest of the discordant samples were considered borderline. Even after applying ddPCR to these samples, discordance still occurred [26].

qPCR has also been used to detect MRD using other markers. For example, when comparing *SOX11* and *CCND1* expression among MCL and healthy donor cohorts, MRD detection using qPCR on isolated mononuclear cells based on *SOX11* expression mirrored the clinical disease development among MCL patients [37]. *SOX11*, a member of the sex-determining region Y-related high-motility-group box transcription factor family, has been a marker of interest in many MRD studies in MCL patients as it is highly expressed in MCL tumors [38]. A key study demonstrated that *SOX11* quantification can be a valid MRD marker, possibly equivalent to t(11;14) [39]. In a review comparing 4 possible MRD markers for MCL (*SOX11*, *CCND1*, Bcl-1/IgH, and IgH-VDJ), the expression of *SOX11* and *CCND1* were shown to be highly correlated in a range of samples from diagnosis to complete remission and relapse, even at low MRD levels. However, IgH-VDJ and Bcl-1/IgH assays are generally more sensitive, indicating a need for improved MRD assays to detect *SOX11* or other marker expression [40]. When qPCR was used to compare different markers by sensitivity and quantitative range, *SOX11* was found to have higher specificity and utility as a predictive value for OS and PFS than t(11;14) in MCL, suggesting it to be a useful marker when other markers cannot be utilized [27].

Even so, the gold standard still has its flaws. qPCR is limited as a choice for relative quantification; it needs a reference standard curve, making it less adaptable for immediate, individual patient management [10, 16]. This makes it difficult to reapply both the primers and probes to other samples, reducing the generalizability of use. It is also complex, time-consuming, labor-intensive, and is restricted in providing target quantification for samples that have a low tumor burden below the quantitative range [28]. This shows the sensitivity limit of PCR methods, indicating a need for a more sophisticated detection method of MRD [26].

On a different spectrum, ddPCR allows for quantification of tumor-specific somatic mutations and is based on sample compartmentalization in oil droplets. While incorporating PCR reactions, ddPCR is also able to track circulating tumor DNA (ctDNA), which is later discussed more in depth [7]. ddPCR therefore differs from traditional PCR methods in that nucleotide particles are segregated into various wells as droplets, giving rise to tens of thousands of droplets [7]. This technique has become quite popular in detecting mutations of interest at baseline in DLBCL and has a detection rate of 10^-5^ [1]. When ddPCR was compared with qPCR among multiple myeloma, MCL, and FL patients via patient-specific IgH rearrangements, ddPCR showed comparable sensitivity, accuracy, and reproducibility to the gold standard [16]. Additionally, unlike qPCR, this method does not require a standard curve as it allows absolute quantification even in samples with no baseline determination of tumor infiltration and ensures quantitative discrimination in a broad range of target amounts (10^5^ to 10^10^) [16, 28]. ddPCR was used in a multicenter phase II study of bortezomib and rituximab maintenance in MCL patients post-ASCT, using another molecular marker, CCND1 mRNA. Although feasibility was achieved with low CCND1 mRNA being detected in all samples without disease progression, the authors were not able to correlate progression with rising mRNA levels as patients were in remission, and relatively few relapses occurred [41]. In the past few years, the workflow and guidelines for ddPCR have been updated within several European countries. This was initiated from the MRD Network of the Fondazione Italiana Linfomi and later moved to the Euro-MRD consortium. Using this updated guideline, the largest comparison study between qPCR and ddPCR in MCL has been done in four prospective EU-MCL clinical trials, with an additional prospective MFC application in one of these trials. The study mainly focused on low MRD level samples, or below the quantitative range samples (BQR), that were defined by qPCR to investigate if ddPCR is able to reduce the number of samples that fall into this “grey-zone.” The results showed that ddPCR, qPCR, and FC measured MRD in a comparable manner, with at least 0.01% positivity from the BQR samples. However, ddPCR was preferred over qPCR as it was more robust in quantifying MRD positivity between the 0.01 and 0.001% range [28]. A major drawback with the use of ddPCR in comparison with qPCR, however, is its lack of validation as a multi-laboratory standard [16]. Currently, the prognostic relevance of ddPCR is under assessment within the EU-MCL Network [28].

### NGS-based MRD methods

Next-generation sequencing, sometimes referred to as high throughput or deep sequencing, is expected to become a companion diagnostic for a variety of lymphomas in the near future. For B-cell lymphomas, clinical utility of NGS-based MRD detection has been reported for CLL, FL, MCL, and DLBCL [16]. With the use of consensus primers, NGS overcomes the limitations of previous patient-specific methods. Two general classifications exist: IgNGS, which amplifies the IgH gene rearrangements and subsequent sequencing to track tumor clones, and mutNGS, which sequences genomic somatic mutations in B cells, generating a modern platform that combines PCR primers with NGS aspects (Table 1) [11].

ClonoSeq*,* formerly called LymphoSIGHT, is the first and only FDA-cleared assay used for MRD monitoring in any lymphoid cancer. It combines multiplex PCR and NGS techniques that is applied to tumor-enriched samples, resulting in amplified and rearranged IgH (VDJ), IgH (DJ), IgK, IgL, and TCR gene segments. It also uses special primers to amplify genomic regions that allows for total nucleated cell content determination. By identifying and tracking tumor clones in follow-up samples, it can provide a comprehensive landscape of gene rearrangements. This method identifies “dominant” sequences that comprise at least 3% of all sequences in the locus and have a frequency of at least 0.2% in the sample’s nucleated cells. This confirms the presence of MRD on clonal lymphoma cells and these sequences are used for MRD tracking assessment [42]. Initially, ClonoSeq was limited by detection of somatic mutations in targeted primer sequences but with recent improvements in primer designs, sensitivity has improved. By utilizing NGS, this method can detect lymphoma cells at a level of one per one million cells. In posttreatment CLL patients, initial MRD testing with MFC resulted in undetectable MRD. However, when subject to ClonoSeq, 74% and 62% of samples in BM and PB mononuclear cells reached a sensitivity of 10^-6^, respectively. These patients, who were now MRD positive, had an inferior PFS compared with those with an undetectable MRD using ClonoSeq [43]. However, ClonoSeq may overestimate MRD frequencies near the limit of detection, which is mainly based on the amount of input DNA. Both the volume and cellularity of a BM sample can affect the ability of this assay to detect low levels of disease [44]. At present, there is a body of scientific work that has been published using ClonoSeq as their MRD assessment tool and its value as a MRD negativity measure and depth of response is higher than other methods in MCL [44]. In a phase II study that tested, the efficacy of rituximab–bendamustine followed by rituximab–cytarabine in transplant-eligible MCL patients, ClonoSeq was applied to detect IgH rearrangements. Among 15 evaluable patients, 93% achieved MRD negativity upon treatment [29]. When MCL patients treated with lenalidomide and rituximab were followed up for 5 years, a high rate of MRD negativity was also found by using a PB MRD assay via ClonoSeq [30]. ClonoSeq was also highlighted at the annual meeting for American Society of Hematology in 2019. Upon a 2-year examination of MRD detection with ClonoSeq in various lymphoid malignancies, ClonoSeq was found to be highly sensitive in observing a deeper disease response to therapy and thus may be able to aid in both therapeutic decision-making and prognostication [45]. A more recent pooled analysis that involved two trial cohorts of transplant-eligible MCL patients treated with rituximab–bendamustine and rituximab–cytarabine also used ClonoSeq. Only a subset of patients from one of the cohorts had MRD assessment done with samples collected at baseline, post-3 cycles of induction, post-6 cycles of induction, and every 3–6 months thereafter. Upon assessment, it was suggested that this particular regimen achieves high rates of MRD negativity that can persist for years post-ASCT [46]. When ClonoSeq was compared with qPCR in 158 follow-up MCL samples, 82% were concordant between the two methods [31]. When MRD was the primary outcome in a study of MCL patients who received bendatmustine–rituximab induction followed by rituximab ± lenalidomide consolidation, MRD status detected by both ClonoSeq and FC correlated with PFS. While both techniques were feasible in assessing MRD and showed concordant results at the 10^-4^ cutoff, ClonoSeq was more sensitive when adequate material was available [32]. Overall, ClonoSeq was able to reach a sensitivity of 1 × 10^-5^, which is comparable to qPCR, but did not require patient-specific reagents [31].

Another technique that utilizes IgNGS to detect MRD is HashClone, which was developed to provide B cell clonality assessment and MRD monitoring over time. This strategy is comprised of two big steps: first, implementation of an alignment-free prediction method to identify tumor clones. Then, by aligning the rearrangements with a reference database, IgH VDJ regions are identified. This technique was utilized to provide marker assessment at MCL diagnosis and MRD monitoring in a sub-study that involved 5 patients from Fondazione Italiana Linfomi. HashClone was able to identify a set of putative clones from each patient and IgH using the international ImmunoGenetics information system database. At different input genomic DNA amounts (100 or 500 ng), NGS data for MRD monitoring was analyzed with a sensitivity comparable to ASO-qPCR [47].

With a high sensitivity and ability to identify patient-specific target sequences, mutNGS techniques have proven valuable in characterizing novel mutations [7]. Due to the use of library preparation and bioinformatics pipelines, this set of techniques is also referred to as panel-directed NGS [1]. The general method involves tissue interrogation for a panel of single nucleotide variants (SNV), insertions or deletions (indels), and/or chromosomal translocations that frequently occur in a specified disease [1]. However, even with a high sensitivity, this method is limited by the breadth of the genes being interrogated as well as the depth of sequences, making it a difficult method in cases with low allele frequency [1]. Targeted locus amplification selectively amplifies and sequences entire genes via crosslinking physically proximal DNA loci. This technology was successful in identifying novel gene fusions in acute lymphoblastic leukemia, which led to a study that applied it to both MCL and FL baseline samples to increase the rate of success marker screening for MRD. Like other studies, this was comparable to qPCR. Among 17 MCL samples, a novel Bcl-1/IgH breakpoint was identified by this method in all samples, indicating the usefulness of this method among patients who did not have traceable MRD markers [48].

## Liquid biopsy and circulating tumor DNA (ctDNA): the future of mantle cell lymphoma management

**Fig. 3:**
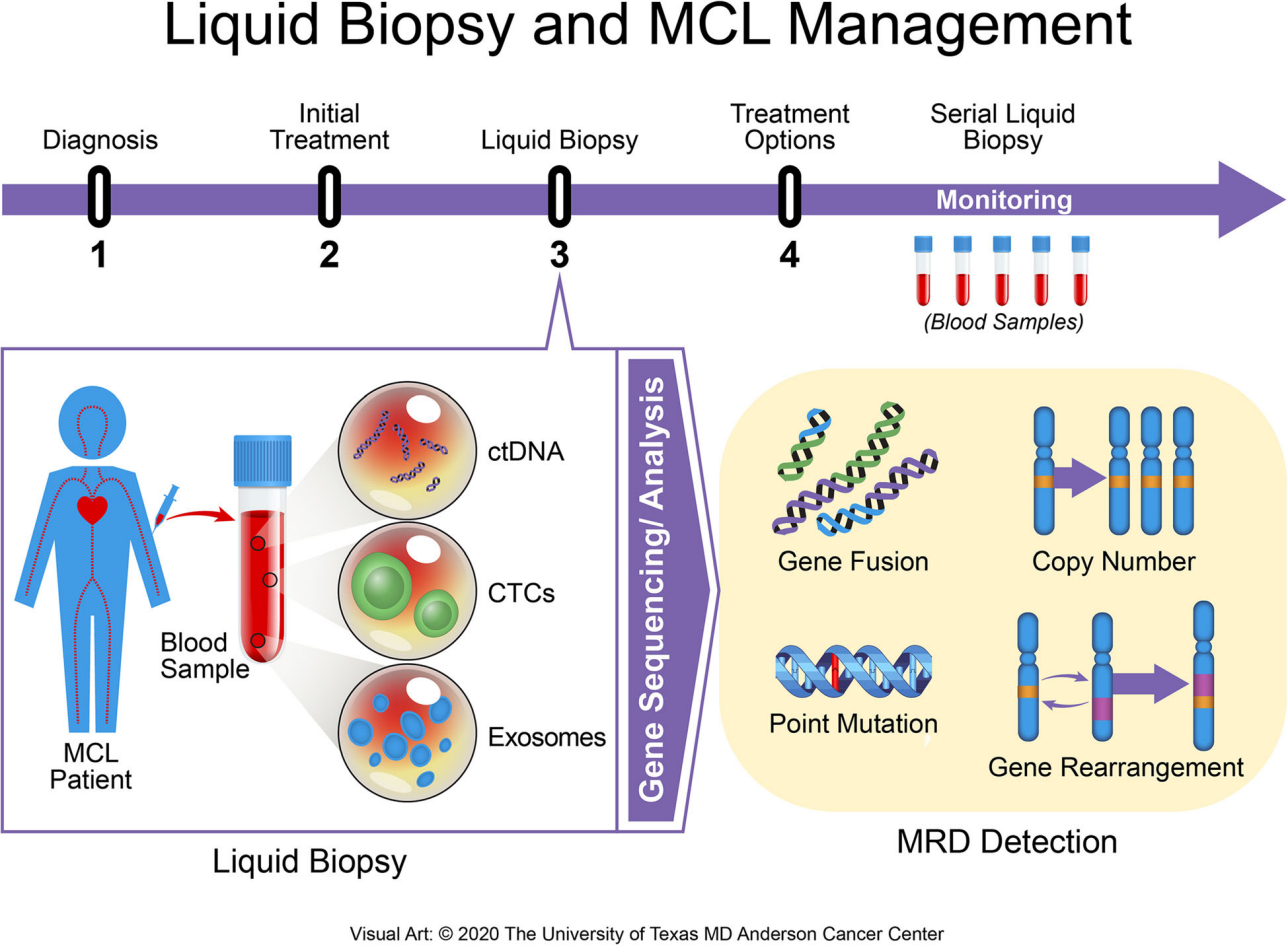
Liquid biopsy application among MCL patients. A timeline of an MCL patient is shown. Upon diagnosis, baseline samples are collected, followed by the initial treatment. A blood sample from the patient is drawn, which includes MRD detectable units: ctDNA, CTCs, and exosomes. The units go through gene sequencing and analysis, where gene fusions, copy number alterations, point mutations, and gene rearrangements can be detected. Once MRD has been detected via liquid biopsy, further treatment options are considered. An additional series of samples are collected during this second treatment to monitor changes in the patient

Although tissue analysis provides a snapshot of what is happening at that moment, tumor evolution at the genetic and molecular level requires more than just a single snapshot to determine the type of therapy needed and to predict the patient’s future path [12]. The term “liquid biopsy” has been coined from the process of integrating a blood draw from patients to evaluate the molecular landscape of the lymphoma. This method is able to acquire all the genetic information from most disease sites, providing both the tumor genotype and a comprehensive picture of its heterogeneity in a noninvasive manner [1, 10, 49]. At the molecular level, the information captured includes point mutations, rearrangements, amplifications, and gene copy variations (Fig. 3) [50]. Liquid biopsies can be conducted using circulating tumor cells, cell-free DNA (cfDNA), ctDNA, exosomes, or other cell-free nucleic acids (mRNA, microRNA) [12]. However, only cfDNA and ctDNA are considered to be practical and feasible for liquid biopsies in most lymphoid malignancies (Table 3).

**Table 3:** Differences in CTC, cfDNA and ctDNA utilization in MRD

	CTCs	cfDNA	ctDNA
Strengths	-Minimally invasive -Easily repeated -FDA-approved method available -Cancer therapy management -Appropriate for solid tumor cancer subtypes	-Minimally invasive -Easily repeated -Easily obtainable -Detect drug mutations -Aid in drug selection and therapy response prediction -Monitoring for emerging drug resistance	-Minimally invasive -Easily repeated -Drug resistance detection at gene level -Driver mutation detection at gene level -Appropriate for liquid tumor cancer subtypes
Weaknesses	-Dilution by benign epithelial cells -Large size not suitable for filtration -Reflects circulating disease only	-Diluted with DNA from healthy cells -Present in inflammatory states and aging -Predetermined somatic alterations needed -Single-cell analysis not permitted	-Standardized nor validated method not available yet -Dilution by cfDNA from non-malignant cells -Low amounts available

cfDNAs can originate from both tumor and non-malignant cells, which can dilute the amount of ctDNA that can be detected in circulation. Nonetheless, cfDNA has been used to track the genetic profile in pre- and post-treatment DLBCL using cancer personalized profiling by deep sequencing (CAPP-seq), another NGS-based method discussed later. cfDNA was comparable to biopsy genotyping that detects somatic mutations and was also able to detect mutations that were far from the biopsy site. Thus, genotyping cfDNA in the plasma can be a strong tool in tracking real-time clonal evolution without being invasive [51]. cfDNA has also been studied as a prognostic biomarker for survival in aggressive NHL (i.e., DLBCL) patients before treatment. Although the median cfDNA concentration in these patients were significantly higher than that of healthy volunteers at 13.7 ng/dl and 7.4 ng/dl, respectively (*p* < 0.001), the cfDNA level was insufficient as an independent prognostic factor, suggesting the need for larger studies [52].

ctDNAs originate from tumor cells that have undergone apoptosis and/or necrosis. It typically comprises < 1% of total cfDNA, is highly tumor-specific, and has a general short half-life that ranges between 16 min to 2.5 h in the blood. Unfortunately, the exact mechanism behind both the release and clearance of ctDNA is not yet fully understood [53]. There are two general approaches for detecting ctDNA. In the targeted approach, mutations are detected in a predefined set of genes and is used in technologies such as AmpliSeq, CAPP-seq, and SFE-SeqS. In the non-targeted approach, de novo mutations can be found by screening the genome without aberrations being a priori (i.e., whole genome sequencing, exome sequencing). Because of these differences, the targeted approach generally has a higher sensitivity than the non-targeted ones [2]. An exception to this is the FoundationACT, a hybrid-capture-based NGS method developed by Foundation Medicine that takes on the broad-based testing approach with no a priori assumptions, allowing detection of both frequent genomic alterations and rare yet important targetable gene drivers [54]. Researchers at Foundation Medicine confirmed that this method reached a 99% sensitivity for short variants at low allele frequencies (< 0.5%). Although the sensitivity decreases with lower allele frequencies, with the lowest being 70% at an allele frequency between 0.125 and 0.25%, this method has been approved and implemented to identify genomic alterations in ctDNA [54].

**Fig. 4:**
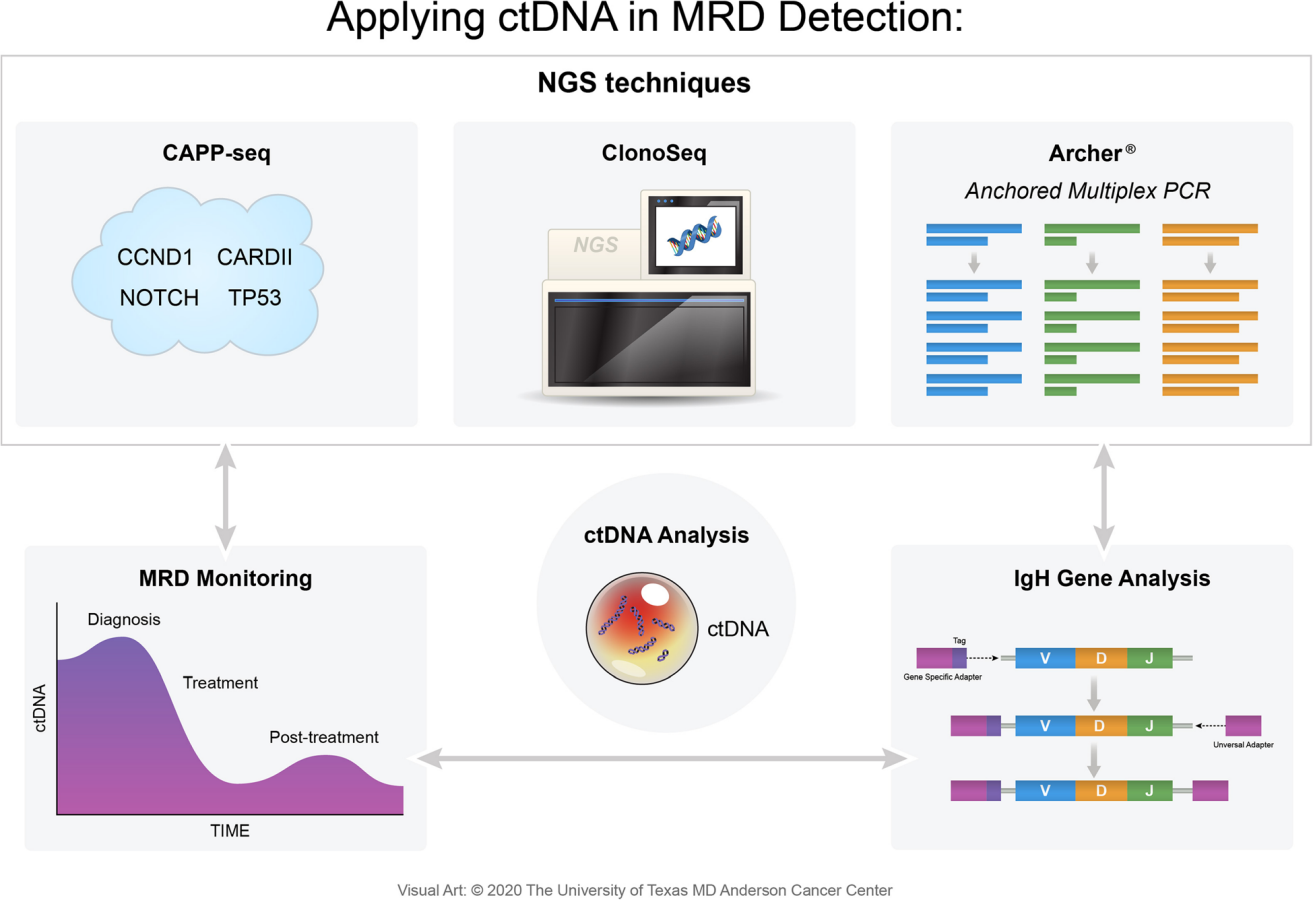
Next-generation sequencing and ctDNA. The detection of plasma ctDNA IgH rearrangements is a valuable tool in MRD assessment. NGS techniques, such as CAPP-seq, ClonoSeq, and Archer, are able to detect these rearrangements with high sensitivity. This allows continuous monitoring of MRD in a patient

The IgNGS approach is also able to detect MRD from peripheral blood mononuclear cells and plasma samples using ctDNA, enabling biopsy-free lymphoma cell detection (Fig. 4) [1]. When lymphoma patients who received hematopoietic stem cell transplantation (HSCT) went under ctDNA-based sequencing via IgNGS techniques, the presence of IgH gene rearrangements were associated with an increased risk of relapse. The clonotype identification rate was 91%, which rendered it readily applicable to patients undergoing HSCT. While IgNGS imposes many strengths, detecting ctDNA does not identify the site or extent of relapse and is not applicable in patients with no identified clonotype and also involves inherent complexities at a high cost, making it not valid in every setting [1, 16].

At the heart of mutNGS is Archer®. Anchored Multiplex PCR (AMP) is a patented target enrichment chemistry engineered to produce sequencer-ready NGS libraries from low input, highly fragmented, or damaged nucleic acid templates, such as formalin-fixed paraffin-embedded samples or cfDNA (Table 4). AMP provides a superior method for enriching specific genetic sequences by utilizing a pool of single gene-specific primers where the template must have both opposing primer binding sites intact for PCR amplification. Prior to PCR amplification, template molecules (i.e., ctDNA) are directly ligated using adapters to incorporate unique molecule barcoding and a synthetic universal priming sequence to enable single-GSP target enrichment, bidirectional target coverage, and sequencing into unknown regions (i.e., fusions and large deletions). Archer’s protocol increases the sensitivity in detecting low-allelic mutations in hematologic samples and because these adapters are ligated to ctDNA fragments prior to PCR, every unique template molecule can be identified, allowing detection of allelic frequencies < 1%. Blood cancer tests combine both FusionPlex® and VariantPlex® to characterize gene fusions, copy number variations, and other variants from a sample [55].

**Table 4:** Differences in ArcherDX and CAPP-seq

	ArcherDX	CAPP-seq
Strengths	-Low input -Anchored multiplex PCR -High sensitivity -Good for low allelic mutations -Fast turnaround time -Easy workflow	-Uses hybridization capture method -Can survey multiple loci at once -Cost effective
Weaknesses	-Not able to survey multiple loci	-Need large input -Prior knowledge of tumor’s genetic landscape -Requires tumor-specific selector -Sample cross contamination -Allelic bias in capture reagent

Developed at Stanford University and currently owned by Roche, the aforementioned CAPP-seq was first validated in solid tumors covering various somatic alterations that resulted in identifying mutations in over 95% of tumors [56]. As a type of ultra-deep NGS method, it utilizes a set of exonic and intronic targets called selectors that are chosen to cover recurrent mutations occurring in a particular cancer. The selected target sequences are then amplified, enriched, and sequenced. By doing so, CAPP-seq captures the complex landscape of somatic mutations of the specific tumor, including SNVs, indels, and breakpoints (Table 4). As a result, it avoids targeting only a single tumor-specific genetic aberration, as is the case with ClonoSeq [57]. Both Archer and CAPP-seq start with trace amounts of ctDNA obtained in a longitudinal fashion from a patient’s plasma, which is then processed and amplified to create libraries. These gene-enriched libraries are then subject to high efficient DNA sequencing, aiding in driver mutation and clonal evolution identifications [54, 56]. Although CAPP-seq is able to combine optimized library preparation and multiphase bioinformatics, a major limitation is the required knowledge of the tumor’s genetic landscape and the need for a tumor-specific selector [56]*.* CAPP-seq showed excellent clinical utility in DLBCL with a true-positive recovery rate of 95–99% and a false-negative rate of 1–5% for confirmed mutations, such as *EZH2*, *MYD88*, and *CD79B* [58]. When CAPP-seq was performed with a panel designed to target 66 genes associated with NHLs on plasma cfDNA, it had a sensitivity of 88% and a specificity > 99% for detecting mutations that are present in a frequency greater than 20% within tumor biopsies [59]. CAPP-seq stands as a highly validated technology, but standardization is still required before putting it into a routine diagnostic procedure for hematological cancers.

Although not yet commercialized, various single-cell genomic-based methods are also applicable in MRD detection in lymphoma. Single-cell genomics, or single-cell sequencing (SCS), takes individual cells to examine sequence information using optimized NGS methods. This provides a higher resolution of the cell and a clearer picture of the individual cell function within its microenvironment. In the context of cancer, SCS has helped in better understanding intratumor heterogeneity, metastasis, and even therapeutic resistance [60]. With developments that involve utilization of unique molecular identifiers, data quality and reproducibility have greatly improved in these SCS methods [61]. Unfortunately, while studies have indicated that SCS methods can aid in providing epimutation information in CLL, not much has been published in regards to MRD assessment in MCL.

Just as there is molecular and cellular heterogeneity in cancer, the amount of ctDNA is highly variable in each cancer subtype [30, 62]. A study that evaluated ctDNA levels across a range of lymphomas using real-time PCR found that the highest levels (up to 2,000 hGE/mL) can be found in mediastinal gray zone and primary mediastinal B cell lymphomas, whereas classical Hodgkin’s and FL had the least amount, up to 20 hGE/mL. Other aggressive lymphomas, such as DLBCL and MCL, had a moderate-to-high amount, somewhere between these two numbers [62, 63]. Therefore, being able to identify any amount of ctDNA, especially those with a very low concentration, is a major and ongoing challenge. This is especially true in regards to early cancer detection, triggering a competitive market for a more sensitive ctDNA technique. While methods such as CAPP-seq have shown promising results, further improvements need to be made, such as being able to analyze large volumes of blood at a time [2, 59]. In a study that evaluated whether ctDNA detection by NGS methods is associated with relapse and survival among lymphoma patients, detectable ctDNA was indeed a predictor for increased risk of relapse and/or progression [64]. However, the applicability was limited to a subset of patients with no clonotype availability, meaning there was no tumor clone frequency of > 5%, for which the authors recommended the evaluation of both peripheral blood mononuclear cells and plasma to reach a higher sensitivity. Using a simple workflow, other approaches have also been considered. The allele-specific PCR methods have been used to detect hot spot mutations, with kits available for clinical use [65]. Cancer hot spot mutations via ctDNA analysis are also the basis for digital PCR on microfluidic platforms, which allows multiplexing. For a larger number of loci, targeted sequencing with PCR amplicons has been used, with molecular barcoding being able to detect allele fractions at a sensitivity below 0.1% (i.e., Archer, CAPP-seq). Using target sequencing methods along with multiplexed patient-specific panels can further heighten the sensitivity [65]. Low specificity is another challenge in the field of ctDNA detection, especially as cancer-associated driver mutations increase with age, regardless of actual cancer development. Somatic mutations in clonal hematopoiesis, a major risk factor for hematologic cancer, were found among 10% of people over the age of 65 who also had a very modest risk of becoming a cancer patient (1% per year). Concomitant detection of these mutations by ctDNA analysis will increase the risk for false positives, and thus decrease specificity [50]. Additions of unique molecular identifiers and digital error suppression can aid in overcoming issues of both false-positive and false-negative results [66].

With the first ctDNA test for *EFGR* mutations being approved, validation of liquid biopsy as a standardized clinical tool for B cell lymphomas is a hopeful advancement [2]. Studies have indicated the importance of choosing the appropriate NGS method when using ctDNA. For example, applying CAPP-seq for ctDNA profiling in DLBCL showed that by simultaneously tracking multiple somatic mutations, it outperforms IgH-based next-generation sequencing methods for the detection of MRD [67]. Pre-treatment ctDNA levels measured by CAPP-seq by serial plasma sampling and molecular responses were also found to be an independent prognostic factor for event-free survival and OS among 217 DLBCL patients in a multicenter study that used a training and validation framework [68]. In this context, pre-treatment ctDNA levels were found to be significantly associated with both IPI and total metabolic tumor volume, which are both prognostic factors in DLBCL. This suggested ctDNA to serve not only as a prognostic factor, but also a quantitative proxy for disease burden. An optimal threshold for ctDNA change was observed and validated, with a 2-log drop at the start of treatment cycle 2 defined as early molecular response and a 2.5-log drop at start of treatment cycle 3 defined as major molecular response. Both responses predicted achievement of complete response. Such early milestone recognition can be useful in areas such as drug development, clinical practice, and a more personalized approach in clinical trial designs [68]. This can also give insight to therapy resistance, a continuing problem in many lymphomas. By analyzing genetic profile changes posttreatment, the resistance mechanism can provide a better therapeutic target among patients [69]. Tracking such mechanisms at relapse has been demonstrated in MCL patients treated with ibrutinib and venetoclax. By exploring the role of ctDNA in real-time monitoring among MCL patients posttreatment, mutations in *ATM* and the switch/sucrose non-fermentable complex were found to be dynamically monitored via ctDNA profiling [70]. Not only that, it was also revealed that when comparing FC and ASO-qPCR to detect IgH rearrangements, ctDNA detection via ddPCR more accurately reflected the treatment response during disease monitoring. Out of the 11 patients who achieved complete response by imaging, which was the primary end point, 10 patients (91%) achieved an MRD-negative status by ctDNA analysis. This was a higher proportion than when assessed by either FC and ASO-qPCR. The study highlighted ctDNA’s ability to not only provide valuable prognostic information but also enable real-time assessment of the treatment by detecting genomic changes that can be associated with resistance [70]. Although the utility of ctDNA assessment can be applied to almost every stage in lymphoid malignancies, there are still needs to be met, such as standardizing ctDNA quantification, using reference standards for each platform, and determining how to integrate ctDNA into risk stratification with established biomarkers (i.e., IPI, interim imaging scans) [71].

## Future implications

Given these MRD detection methods, it is critical to assess whether and how these tools aid in improving the management of MCL patients. At any given moment, measuring the disease burden provides a very in-depth detail of the patient’s progress, whether as a prognosis value or treatment indication [56]. MRD data continues to evolve in MCL, and while routine practice is not yet implemented, ongoing and stronger data will make this possible in the near future. Incorporating MRD assessment as a primary endpoint in clinical studies rather than as an exploratory one is a realistic way data can be strengthened. As seen in Table 2, studies that have MRD as the primary endpoint has shown significant results. Also noteworthy is the higher MRD negative status seen in MCL patients who receive ASCT [6, 22, 23, 33]. This indicates that while intense and not fit for the elderly population, ASCT can be effective in clearing any residual disease. The clinical application of MRD detection is mostly determined by the timing of sample collections: pre-treatment, during treatment, and posttreatment. Monitoring ctDNA levels via liquid biopsy can be especially useful during cancer surveillance upon patients achieving remission. The prognostic information obtained from such monitoring is available for use in clinical trials as a primary or secondary endpoint. Thus, the use of MRD detection pre-treatment provides new opportunities for precision treatment. In a review done by Roschewski and colleagues, a high ctDNA concentration present before treatment has shown to correlate with poor prognosis in DLBCL, leading to the choice of a more aggressive treatment [11]. Once treatment is started, changes in ctDNA levels can provide early feedback on the treatment efficacy in patients with advanced diseases [57]. By monitoring the changes in molecular disease levels, one can understand the changes in tumor burden: high levels with progressed disease and low levels with stable disease [7]. Liquid biopsy is the most utilized technique for mid-treatment MRD detection. The likelihood of recurrent disease following treatment can also be predicted via MRD detection, providing information on further prognosis. Patients with a low disease burden at recurrence have better outcomes, again highlighting the ability for MRD detection methods to capture minimal amount of ctDNA [10]. An ongoing clinical trial that is detecting and quantifying ctDNA in MCL patients throughout therapy have demonstrated earlier detection of relapsed disease using IgNGS [72]. Risk stratification can also be conducted, especially if a patient is to be in a clinical trial. A study based on a population that has been stratified by risk via MRD can further aid in precision treatment.

## Conclusion

While it is true that MRD assessment has evolved over time as both a diagnostic tool and predictor of long-term remission, the ability to personalize and match the appropriate treatment to a patient while dynamically monitoring its progress is still in the beginning stages. Notably, while qPCR is known to be the gold standard, it is easy to recognize that NGS methods have much broader applicability (Table 1). By allowing precision therapy that targets individuals rather than the population, the noninvasive liquid biopsy is thought to be the ultimate MRD detector in hematological cancers. Even still, there are barriers to overcome before MRD assessment can reach its full potential in MCL. First, MRD monitoring is not validated in ongoing clinical trials, especially in trials assessing novel treatment, making it difficult to evaluate its applicability. In order to effectively compare available data, MRD assessments need to be standardized and interlaboratory quality control would be required. The aforementioned study of MRD assessment among MCL patients who have been treated with ibrutinib is a good example on how future clinical trials should amend their protocols [20]. This lack of use can be attributed to the high cost and specificity required to analyze the results, mandating technical expertise. A study analyzing the overall healthcare expenses that specifically impact patients’ quality of life is a good starting point for motivation to decrease the per sample cost of MRD assessment via NGS. Second, there is a lack of a reliable molecular marker for MRD in patients. Although ctDNA levels have proven to be good prognostic markers, more studies are needed as previously indicated. Finally, before NGS-based MRD assessment techniques can be introduced into clinical practice, a large-scale validation study of each of the techniques must be conducted to finalize their peak performance. Furthermore, liquid biopsy technologies are regulated by agencies such as the FDA and the European Medicine Agency, so both the safety and effectiveness criteria must be fulfilled before being approved for clinical use. Being able to provide accurate prognosis through proper MRD detection can aid in selecting the most appropriate treatment of care followed by continuous monitoring of a patient’s progression via interim MRD detections. While larger clinical studies that can validate MRD detection to be an independent prognostic factor is still in need, there is imminent hope for MRD-driven treatment approaches to become the standard protocol on the road to curing MCL.

## Data Availability

Not applicable

## Abbreviations

MRD: Minimal residual disease
PB: Peripheral blood
BM: Bone marrow
CLL: Chronic lymphocytic leukemia
NHL: Non-Hodgkin’s lymphoma
DLCBL: Diffuse large B cell lymphoma
FL: Follicular lymphoma
MCL: Mantle cell lymphoma
FC: Flow cytometry
PCR: Polymerase chain reaction
NGS: Next-generation sequencing
qPCR: Real-time quantitative PCR
IgH: Immunoglobulin heavy chain
MFC: Multi-parameter flow cytometry
ddPCR: Digital droplet PCR
OS: Overall survival
PFS: Progression-free survival
R-CHOP: Rituximab, cyclophosphamide, doxorubicin hydrochloride, vincristine sulfate, prednisone
ASO-qPCR: Allele-specific oligonucleotide qPCR
ASCT: Autologous stem cell transplant
R-DHAP: Rituximab/dexamethasone/cytarabine/cisplatin
MIPI: MCL international prognostic index
*SOX11*: Sex-determining region-box transcription factor 11
*CCND1*: Cyclin D1
ctDNA: Circulating tumor DNA
BQR: Below the quantitative range samples
SNV: Single nucleotide variants
indel: Insertions or deletions
cfDNA: Cell-free DNA
CAPP-seq: Cancer personalized profiling by deep sequencing
AMP: Anchored multiplex PCR
SCS: Single-cell sequencing
EFGR: Epidermal growth factor receptor
ATM: Ataxia-telangiectasia mutated
